# BH4-Mediated Enhancement of Endothelial Nitric Oxide Synthase Activity Reduces Hyperoxia-Induced Endothelial Damage and Preserves Vascular Integrity in the Neonate

**DOI:** 10.1167/iovs.16-20523

**Published:** 2017-01

**Authors:** Kevin S. Edgar, Orla M. Galvin, Anthony Collins, Zvonimir S. Katusic, Denise M. McDonald

**Affiliations:** 1 Centre for Experimental Medicine, Queen's University Belfast, United Kingdom; 2 Department of Anesthesiology and Pharmacology, Mayo Clinic, Minnesota, United States

**Keywords:** endothelial nitric oxide synthase, retinal vasculature, retinopathy of prematurity

## Abstract

**Purpose:**

Endothelial nitric oxide synthase (eNOS)-derived nitric oxide (NO) has important vasoprotective functions that are compromised in the vasodegenerative phase of retinopathy of prematurity, owing to hyperoxia-induced depletion of the essential NOS cofactor BH4. Because modulating eNOS function can be beneficial or detrimental, our aim was to investigate the effect of BH4 supplementation on eNOS function and vascular regression in hyperoxia.

**Methods:**

Endothelial-specific eNOS-green fluorescent protein (GFP) overexpressing mice at postnatal day 7 (P7) were exposed to hyperoxia for 48 hours in the presence or absence of supplemental BH4, achieved by administration of sepiapterin, a stable BH4 precursor. Tissue was collected either for retinal flat mounts that were stained with lectin to determine the extent of vessel coverage or for analysis of BH4 by high-performance liquid chromatography, nitrotyrosine (NT) marker by Western blotting, VEGF expression by ELISA, and NOS activity by arginine-to-citrulline conversion. Primary retinal microvascular endothelial cells (RMEC) were similarly treated, and hyperoxia-induced damage was determined.

**Results:**

Sepiapterin effectively enhanced BH4 levels in hyperoxia-exposed retinas and brains, elevated NOS activity, and reduced NT-modified protein, leading to reversal of the exacerbated vasoregression observed in the presence of eNOS overexpression. In RMECs, hyperoxia-mediated depletion of BH4 dysregulated the redox balance by reducing nitrite and elevating superoxide and impaired proliferative ability. BH4 supplementation restored normal RMEC proliferation in vitro and also in vivo, providing a mechanistic link with the enhanced vascular coverage in eNOS-GFP retinas.

**Conclusions:**

These results demonstrate that BH4 supplementation corrects hyperoxia-induced RMEC dysfunction and preserves vascular integrity by enhancing eNOS function.

Retinopathy of prematurity (ROP) is a sight-threatening complication of premature birth that is compounded by the therapeutic hyperoxia that is essential for the survival of premature infants.^1,2^ Development of the inner retinal circulation occurs in the third trimester of pregnancy; therefore, when infants are born prematurely, this normal retinal vascular development is stunted. The hyperoxic environment causes further regression of the pre-existing immature vessels (phase 1). Thus, when oxygen is removed, the resulting vascular insufficiency leads to irreversible ischemia-induced tissue damage and hypoxia-induced intravitreal neovascularization (NV; phase 2), which, if left untreated, leads to vision loss.^1,2^ Importantly, there is a strong correlation between the extent of avascularity in phase 1 and severity of NV in phase 2, in which infants born very prematurely have larger areas of ischemia and more severe retinal neovascularization.^3,4^ This suggests that treatments that could preserve endothelial cell (EC) integrity during hyperoxia and support normal vascular development could reduce the need for treatments such as pan retinal laser photocoagulation and intravitreal injections of anti-VEGF agents, which focus on late disease and carry significant risk of serious complications.^5,6^ Mechanistically, a significant amount of research indicates a central role for oxidative stress in the initial cell injury in ROP and subsequent vasoregression.^7–10^ In this regard, we and other investigators have previously shown that the nitric oxide (NO)-producing enzyme endothelial NO synthase (eNOS) plays an important role in such oxidative injury to the immature retinal vasculature.^7,10,11^ More specifically, using a loss of function transgenic model, Brookes et al.^7^ showed that reducing peroxynitrite levels by eNOS depletion in eNOS knockout animals reduced capillary dropout and enhanced vascular coverage following hyperoxia, demonstrating a role for eNOS-derived peroxynitrite in promoting vascular regression. At the other extreme, using a gain of function transgenic model with augmented endothelial-specific eNOS expression (eNOS-green fluorescent protein [GFP] transgenic animals), we showed that eNOS is dysfunctional in hyperoxia and acts as a source of the oxygen free radical superoxide (O_2_^−^) instead of its normal vasoprotective product, NO, dysregulating the cellular redox balance and exacerbating retinal vascular regression.^10,12–14^ In a separate study, we showed that hyperoxia depletes levels of the NOS cofactor tetrahydrobiopterin (BH4) in the neonatal retina, resulting in eNOS uncoupling and a shift from NO to O_2_^−^.^10,15^ This eNOS dysfunction was reversed by supplementing ex vivo tissue homogenates with BH4.^10^ Together, these findings indicate that, in hyperoxia, there is a nonstoichiometric relationship between active eNOS and BH4 for optimal NO production and suggests that correction of the discrepancy could translate to improved eNOS-mediated vascular preservation in vivo.^16,17^ Manipulating eNOS activity can have either beneficial or detrimental consequences, especially in a pro-oxidant environment such as hyperoxia. In such situations, the presence of reactive oxygen species (ROS) would negate the positive impact of NO and even exacerbate vascular damage through the reaction of ROS and NO, leading to higher levels of relatively long-lived peroxynitrite levels and exacerbating outcomes for the vasculature. Thus, here our aim was to determine whether supplementing BH4 levels in vivo could reverse the impaired eNOS function and protect the retina from hyperoxic insult. Our working hypothesis was that improving BH4 levels to compensate for its oxidative loss in elevated oxygen would diminish hyperoxia-induced vascular regression by normalizing the cellular redox balance. In order to manipulate the relative levels of eNOS and BH4, we used neonatal animals overexpressing eNOS specifically in the vascular endothelium and treatment with sepiapterin to increase BH4.

## Materials and Methods

### eNOS-GFP Animals and Hyperoxia Treatment

Animals overexpressing eNOS-GFP (a kind gift from Professor Rini de Crom, Department of Cell Biology and Genetics, Erasmus University Medical Centre Rotterdam, Rotterdam, The Netherlands) on a C57/BL6 background were used. These animals carry the full-length human genomic DNA sequence, including the endogenous eNOS promoter, fused in frame with an enhanced GFP (eGFP) reporter sequence, allowing expression of a functional eNOS-GFP fusion protein, as described previously.^10,18^ Heterozygote eNOS-GFP-positive animals and their wild-type (WT) homozygous littermate controls were used throughout. All animal studies were performed under a project license issued by the UK Home Office (Animals Scientific Procedures Act 1986), approved by the local animal care ethics committee, and conducted in accordance with the ARVO statement for the use of Animals in Ophthalmic and Vision Research. The in vivo protocol was based on the early hyperoxia-induced vaso-obliterative (VOB) stage of the murine oxygen-induced retinopathy (OIR) model.^19^ According to this protocol, neonatal mice and their nursing dams are exposed to 75% oxygen between postnatal day 7 (P7) and P9 to P12, a time frame when the primary inner plexus of the retinal vasculature is complete but still vulnerable to hyperoxic damage. The resulting EC dysfunction leads to regression of pre-existing retinal vessels, evident as large areas of avascular central retina.^7,10,15^ Thus, for all in vivo studies, WT and eNOS-GFP transgenic animals were exposed to 75% oxygen from P7 to P9 before tissues were collected for processing.

### Retinal Flat Mount Processing and Vascular Phenotype

Paraformaldehyde (PFA)-fixed eyes were flat mounted, and blood vessels were labeled with biotinylated isolectin (20 μg/mL; Sigma-Aldrich, Gillingham, UK), followed by streptavidin Alexa Fluor-405 or 568 conjugate (Life Technologies Ltd., Paisley, UK), as described previously.^10,15^ Lectin-stained flat-mounted retinas were imaged using a fluorescence microscopy (Eclipse E400 model; Nikon Corporation, Kingston upon Thames, UK), and vascular coverage was measured using NIS Elements BR software (Nikon Corporation).

### Biopterin Analysis by HPLC

Tissue BH4 and BH2 (an oxidation product of BH4 that competitively inhibits the catalysis of NO conversion) were measured, along with standards, by high performance liquid chromatography (HPLC) with fluorescence detection after iodine oxidation in acidic or alkaline conditions as described previously.^15,20,21^ BH4 concentrations, expressed as nanograms per milligram of protein, were calculated by subtracting BH2 plus oxidized biopterin from total biopterin.

### In Vivo Supplementation Protocol

Supplementation with BH4 was achieved by the addition of sepiapterin, a stable synthetic pterin converted intracellularly to BH4 through the pterin salvage pathway, as described previously.^22–25^ At P7, mice were assigned to the control or treatment group. Wild-type and eNOS-GFP transgenic animals were supplemented with BH4 and subjected to 75% oxygen from postnatal days 7 to 9. Treatment was administered by intraperitoneal injection, 10 mg/kg sepiapterin dissolved in dimethyl sulfoxide (DMSO), based on the manufacturer's recommendations, with control animals receiving an equivalent amount of DMSO diluted in sterile saline solution (vehicle control [VC] of 3.5% [v/v] or 0.35 μL/g DMSO). This sepiapterin concentration was based on evidence from other studies that this dose is well tolerated and results in elevation of tissue BH4 level that produce physiologically measurable effects.^23–25^ Following 48 hours exposure to 75% oxygen, eyes were collected and fixed for lectin staining, or retinal and brain tissue were collected and immediately snap frozen on dry ice and stored at −80°C for later analysis.

### NOS Activity Assay

Nitric oxide in tissue samples was measured by NOS activity by the conversion of radiolabeled arginine-to-citrulline, using a modified NOS activity assay in the absence of additional assay BH4 and in the presence of an arginase inhibitor, as described previously (Cayman Chemical, Ann Arbor, MI, USA).^15,26^ For each sample, background was determined by incubation in the presence of N-Nitro-L-arginine (L-NNA) and subtracted from the total counts. A portion of the sample was used for Western blotting.

### Western Blotting

Nitrotyrosine (NT) assay Western blotting was conducted as previously described.^15,26^ Equivalent amounts of protein sample, typically 30 μg, were separated on a 9% SDS-polyacrylamide gel, transferred to polyvinylidene fluoride membrane (Millipore, Watford, UK) and immunostained with anti-eNOS, iNOS, nNOS (BD Biosciences, Oxford, UK), or monoclonal anti-NT (Cayman Chemicals) primary antibodies, followed by the appropriate horseradish peroxidase-conjugated secondary antibody (Insight Biotechnology Ltd., London, UK). β-Actin mouse monoclonal antibody (Sigma-Aldrich) was used to verify equivalency of loading.

### VEGF ELISA

Retinas were pooled and homogenized in phosphate-buffered saline (PBS) containing protease inhibitors, and VEGF levels were determined by ELISA (R&D Systems, Minneapolis, MN, USA), as described previously.^15^

### Dopamine ELISA

Pooled retinal samples were homogenized in lysis buffer (50 mM Tris-HCl, pH 7.5, 150 mM NaCl, and 1% nonionic nondenaturing detergent [IGEPAL CA-630]) containing 10 mM ascorbic acid and 0.1 mM EDTA and assayed for dopamine, using an ELISA kit (catalog number BA E-5300; Labor Diagnostika Nord, Nordhorn, Germany), as described previously.^15^

### Primary Retinal Microvascular Endothelial Cell (RMEC) Culture and Measurement of Cellular BH4 Levels

Primary retinal microvascular ECs (RMEC) were isolated and cultured from bovine eyes, as described previously, and routinely grown on gelatin-coated tissue plates (1%; Sigma-Aldrich).^27^ Hyperoxia treatment was performed by maintaining cells in 75% oxygen/5% CO_2_, using a gas controller (Proox model C21 and C-Chamber incubator insert; BioSpherix, Lacona, NY, USA).

### In Vitro BH4 Supplementation

RMEC (80,000 cells per well) were seeded onto 1% gelatin-coated 6-well plates and, after 24 hours, when cells were approximately 50% confluent, were incubated in growth medium containing vehicle control (1:10,000 dilution of DMSO), 0.1μM sepiapterin (dissolved in DMSO; Sigma-Aldrich), or 1 μM sepiapterin and placed under hyperoxic or normoxic conditions for 24 hours. For BH4 and BH2 measurements, cells were collected by trypsinization, and the resulting cell pellet was snap frozen on dry ice and stored at −80°C until analysis. Briefly, cell pellets were homogenized in cold extraction buffer (50 mM Tris-HCl, pH 7.4, 1 mM dithiothreitol [DTT], 1 mM ethylenediaminetetraacetic acid [EDTA]; all Sigma-Aldrich) and centrifuged at 14,000 rpm for 15 minutes at 4°C. BH_4_ levels were determined by HPLC with fluorescence detection after iodine oxidation under acidic or alkaline conditions, as described above and previously.^15^ Parallel plates were used to measure superoxide detection by dihyroethidium (DHE) or proliferation by 5-ethynyl-2′-deoxyuridine (EdU).

### Nitrite Measurements

Nitric oxide was measured by accumulation of the stable by-product nitrite by using the Griess assay.^27^ Briefly, RMEC, seeded onto 24-well plates, were treated with sepiapterin in phenol red-free Dulbecco's modified Eagle medium (Life Technologies Ltd.) in the presence or absence of the NOS inhibitor N-Nitro-L-arginine methyl ester (L-NAME, 1 mM; Sigma-Aldrich) and exposed to normoxic and hyperoxic conditions for 24 hours. Following treatment, medium was removed and centrifuged before addition of Griess reagent, and sample absorbance was detected at 540 nm (FLUOstar Omega; BMG Labtech, Aylesbury, UK).

### EdU Assay

Proliferation was assessed in RMEC by EdU incorporation, using the Click-iT EdU imaging kit (Life Technologies). RMEC (80,000 cells per well) were seeded onto gelatin-coated glass cover slips and allowed to attach overnight. Cells were then treated with sepiapterin or VC and exposed to 75% oxygen (with normoxic controls) in the presence of EdU (10 μM). Following incubation, cells were fixed with 3% PFA for 15 min at room temperature, rinsed twice with blocking buffer (3% BSA; Sigma-Aldrich) in PBS, and then permeabilized with 0.5% Triton X-100 in PBS for 20 minutes. Cells were washed twice more and stained with Alexa-Fluor-555 fluorescent nuclear dye marker (Life Technologies) and Hoechst 33342 dye (Life Technologies) before mounting with Vectashield (Vector Laboratories Ltd., Peterborough, UK). Nonoverlapping images were collected for each treatment (4 images each from triplicate wells) by using inverted confocal laser scanning microscopy (TE2000-U; Nikon Corp.), and the percentage of EdU-positive cells, as the percentage of the total cell number, was determined by Hoechst 33342 dye staining, using EZ Freeviewer version 3.9 software (Nikon Corp.).

### DHE For Analysis of Superoxide Production

In situ free radical production was assayed using the oxidative fluorescent dye DHE, in the presence and absence of the inhibitors PEG-SOD and L-NAME.^27^ Cells were treated as described above, rinsed in phenol red-free Dulbecco's modified Eagle medium, incubated with 5 μM DHE (Life Technologies) diluted in the same medium, and exposed to normoxic and hyperoxic conditions for 24 hours. Cells were also reacted with DHE in the presence of L-NAME (1 mM) and PEG-SOD (100 U/mL; Sigma-Aldrich). Three images were collected per well from 6 wells per treatment, using inverted confocal laser scanning microscopy, with identical acquisition settings for comparison of intensities.

### BrdU Labeling of Hyperoxia-Treated P9 Pups and Branch Point Analysis

For in vivo analysis of proliferation, mice were injected intraperitoneally with 300 μg of 5′-bromo-2′ deoxyuridine (BrdU; Life Technologies), 3 hours before being euthanized on P9 following supplementation. Eyes were fixed in 4% PFA-PBS for 30 min at room temperature and washed with PBS before being incubated with formamide-SSC (NaCl and sodium citrate) solution for 1 hour at 65°C. Retinas were then incubated in 6 N HCl solution for 30 min at 37°C and then neutralized by washing with 0.1 M Tris-HCl (pH 8.0). Specimens were treated with blocking buffer, then incubated overnight at 4°C with anti-BrdU antibody (Dako, Ely, UK), and washed again. Secondary goat anti-mouse antibody (Alexa Fluor 546; Life Technologies) was added before mounting. Images were obtained at 400× magnification. The number of BrdU-positive nuclei within the lectin-stained vascular area was quantified and expressed per retinal quadrant. Branching was also determined from the same sections as previously described.^10,15^

### Statistical Analysis

Statistical analysis was performed using Prism version 5.03 software (GraphPad Software, La Jolla, CA, USA) by independent Student's *t*-test to determine differences between the two groups (*P < 0.05; **P < 0.01; ***P < 0.001). When comparisons between more than two groups were performed, 1-way ANOVA with Bonferroni's multiple comparison test was used to determine statistical significance. Data are means ± SEM from 3 to 6 individual litters. For in vitro studies, all experiments were performed using at least 3 independent isolations and are the results of 3 to 4 independent experiments, unless otherwise indicated.

## Results

### Effect of Hyperoxia on Vascular Regression and Retinal BH4 Levels

**Figure 1:**
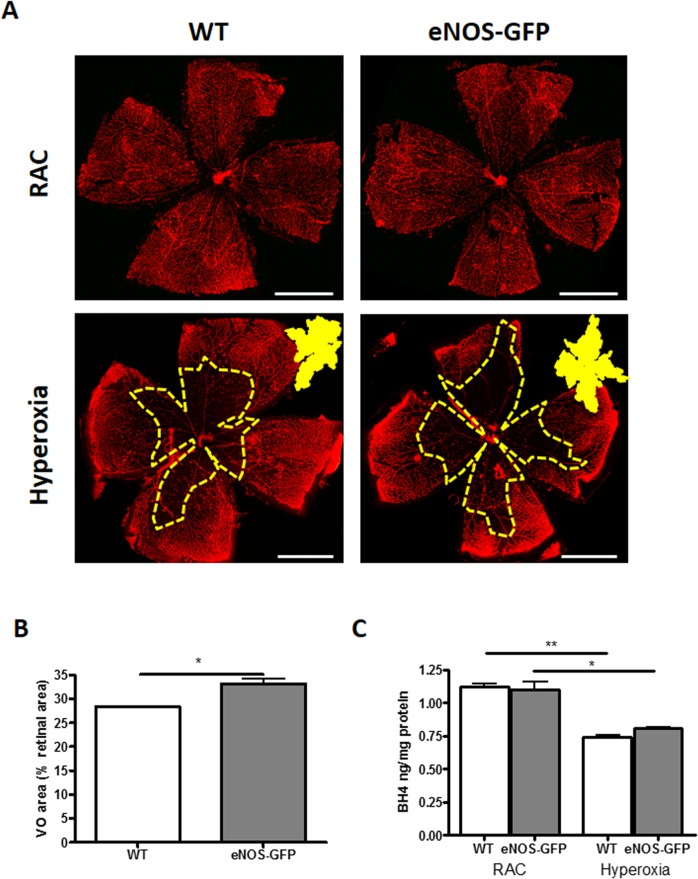
Hyperoxia causes vascular regression and depletes BH4 in vivo. Hyperoxia exposure of mouse pups from P7 to P9 results in a marked vascular regression and depleted retinal BH4 levels. (A) Representative images of room air controlled and hyperoxia-exposed animals. Hyperoxia-induced vascular regression in the central retina (yellow) is significantly enhanced in eNOS-GFP animals compared to their WT litter mate controls. (B) Avascular or VO area is expressed as a percentage of total retinal area. (C) Hyperoxia exposure of mouse pups results in a marked reduction in retinal BH4 levels measured by HPLC. *P < 0.05, **P < 0.01.

Hyperoxia-induced vascular regression was observable as a vessel-free zone in the central retina in neonatal pups at P9 after 48 hours' hyperoxia (Fig. 1A). Previously, we showed that exposure to 5 days of hyperoxia in eNOS-GFP mice exacerbated vaso-obliteration compared to their WT counterparts.^10^ Here, using the shorter exposure of 48 hours to elevated oxygen, we showed that vascular regression at P9 was comparable to that previously seen at P12 (Figs. 1A, 1B). Importantly, the exacerbated regression previously reported in the eNOS-GFP group was also evident at P9 (28.4% in WT mice) and 33.2% (eNOS-GFP mice). Comparison of BH4 levels between P9 room air controlled and hyperoxia-treated P9 retinas showed a significant decrease (almost 30% reduction) in BH4 levels (Fig. 1C).

### Efficacy of Sepiapterin to Elevate Retinal BH4 Levels and Effect of Supplementation on Hyperoxia Between P7 and P9

**Figure 2:**
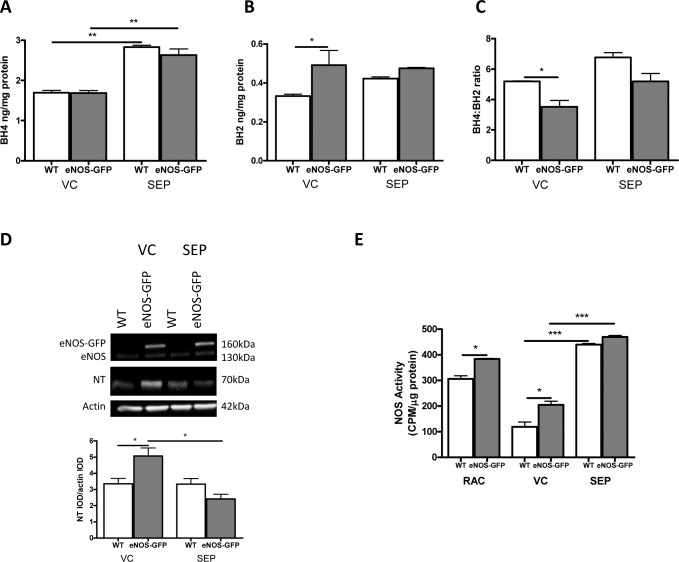
Sepiapterin supplementation increases retinal BH4 levels and improves NOS enzymatic activity, which correlates with a reduction in oxidative stress. eNOS-GFP and WT littermates were injected with VC or sepiapterin at P7 before being exposed to hyperoxia for 48 hours. (A–C) Sepiapterin supplementation caused a significant increase in retinal BH4 level in both WT and eNOS-GFP mice. Histogram shows representative data from 1 of 3 experiments. (D) NT protein level was measured by Western blot analysis as an indicator of retinal oxidative stress. Following treatment with BH4, there is a significant reduction in the level of NT-modified protein in eNOS-GFP mice. Total eNOS protein in the same sample assayed for NT shows the eNOS protein level in VC is equivalent to that in the sepiapterin group. (E) Analysis of NOS activity showed a significant increase in NOS activity in supplemented mice compared to that in controls. *P < 0.05, **P < 0.01, ***P < 0.001.

Next, we investigated whether supplementing retinal BH4 levels could reverse the hyperoxia-mediated defect in BH4 levels and prevent vascular regression. BH4 is easily oxidized, therefore, BH4 supplementation was achieved by addition of sepiapterin, which has previously been shown to raise tissue BH4 levels more efficiently than BH4 in mice.^23^ We first confirmed the ability of sepiapterin to increase BH4 in the retina and demonstrated that increased levels were detectable 48 hours after injection in brain and retinal tissue samples following hyperoxia (Fig. 2A, Supplementary Fig. S1). Sepiapterin supplementation of mice at P7 and subsequent hyperoxic exposure for 48 hours resulted in a significant elevation of BH4 levels in retinal (Fig. 2A) and brain (Supplementary Fig. S1) tissue: BH4 increased by 67% in WT and 55% in eNOS-GFP mice in retinal samples, demonstrating no effect of genotype. BH2 is an oxidation product of BH4 that competes with BH4 and reduces the ability of eNOS to produce NO; accordingly, studies have suggested that the BH2:BH4 ratio is more predictive of eNOS dysfunction than the absolute concentration of BH4. Here, in contrast to the BH4 levels, which were similar across genotypes, there was a measurable difference in BH2 (Fig. 2B) in the eNOS-GFP group compared to that in WT VC-treated controls; this resulted in a decrease in the level of BH4 relative to that of BH2, suggesting some oxidation (Fig. 2C) in the presence of eNOS overexpression. Importantly, this was normalized by sepiapterin, in correspondence with the reversal by sepiapterin of the hyperoxia-associated decrease in NOS activity (Fig. 2E). Hyperoxia reduced NOS activity by 61% and 47% in WT and eNOS-GFP, respectively, compared to room air-treated controls. In eNOS-GFP animals, the hyperoxic reduction in NOS activity was paralleled by an increase in NT immunoreactivity (Fig. 2D), demonstrating increased peroxynitrite formation compared to that in WT mice. These findings, together with the higher BH2 in the eNOS-GFP group, indicated higher generation of peroxynitrite and peroxynitrite-mediated BH4 oxidation. Sepiapterin treatment reduced this retinal NT immunoreactivity (Fig. 2D) and reversed the hyperoxia-mediated defect in NOS activity (Fig. 2E) in the eNOS-GFP group, confirming a BH4-dependent impairment in NOS function in hyperoxia. Expression of eNOS-GFP fusion protein or endogenous eNOS was unaffected by treatment (Fig. 2D). Together, these findings are indicative of improved NO availability and decreased oxidative free radical production following supplementation. In contrast to the augmented eNOS group, in the WT group the effect of sepiapterin on the level of NT was minimal. Notably, however, this correlated with raised VC-treated BH4 levels compared to the control group shown in Figure 1; 0.75 ng/mg protein in the hyperoxia treated group (Fig. 1) was raised to 1.7 ng/mg protein in the VC group (Fig. 2), suggesting that vehicle alone had some antioxidant effects. This effect was also evident in the lower NT immunoreactivity of the WT VC group; supplementation had no further measurable effect on NT levels.

BH4, in addition to being a cofactor for eNOS, is a cofactor for the other NOS isoforms, inducible and neuronal NOS, and is also a cofactor for the aromatic hydroxylases. Here, retinal dopamine levels were quantified following sepiapterin supplementation in room air-control animals and showed an elevation in dopamine levels that was similar for both genotypes (Supplementary Fig. S2A). In contrast, in the same experimental groups, the BH4-mediated rise in NOS activity was much more pronounced in the eNOS-GFP group than in the WTs (Supplementary Fig. S2B). Together, this demonstrated that NO production and not dopamine was differentially enhanced by the presence of additional eNOS. Thus, any differences in vascular responsiveness between the groups (WT versus eNOS-GFP) is due to endothelial eNOS overexpression. Expression levels of eNOS, nNOS, and iNOS were also determined in the same samples (Supplementary Fig. S2C). ENOS expression was elevated in the eNOS-GFP groups as expected, iNOS was not present, and nNOS showed a small increase in expression in both the WT and eNOS-GFP groups after sepiapterin supplementation. Because the expression levels were similar between the two genotypes, any differences in effect of genotype on total NOS activity were considered likely to be mediated by the presence of the eNOS-GFP transgene.

### Effect of BH4 Supplementation on Hyperoxia-Induced Vascular Regression in the Presence of eNOS Overexpression

**Figure 3:**
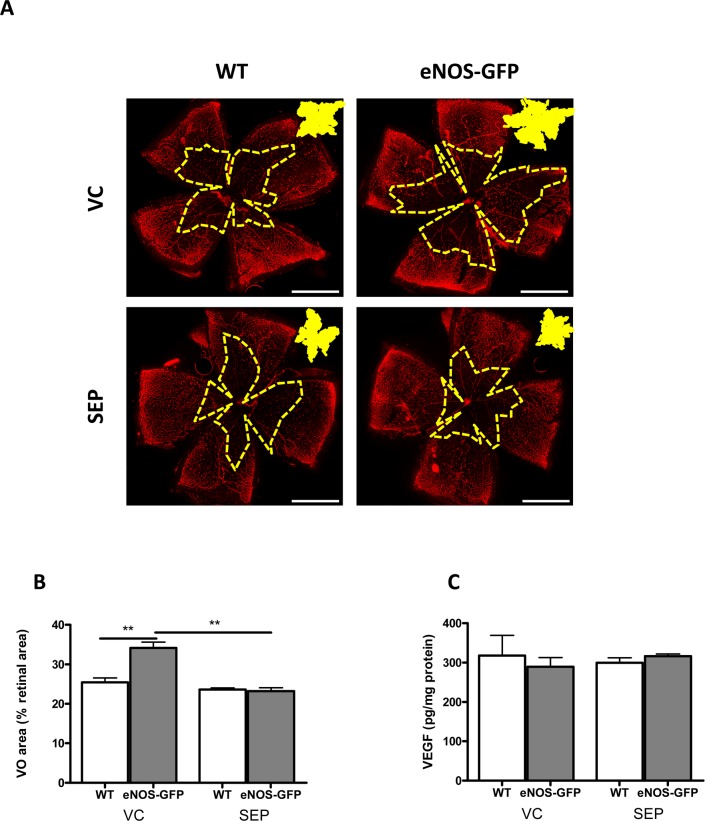
Sepiapterin administration reduces vascular regression following hyperoxia exposure in eNOS-GFP mice. (A) Vaso-obliteration following hyperoxia exposure in eNOS-GFP mice is reduced following sepiapterin administration. Mice were injected at P7 prior to being placed into a hyperoxic chamber. (B) Avascular area or VO area, shown as a percentage of total retinal area, in eNOS-GFP mice was significantly higher than that in WT mice. Supplementation with sepiapterin caused a significant decrease in VO area in eNOS-GFP mice. (C) VEGF was measured by ELISA in pooled retinal sample lysate following sepiapterin supplementation. Scale bars: 500 μm; n = 3 litters per group. **P < 0.01.

In the WT group, BH4 supplementation produced a marginal decrease in vascular closure compared to VC (Fig. 3A, 3B) from 25.4% avascular area in the VC to 23.6% in the sepiapterin group. Notably, compared to the uninjected controls shown in Figure 1, this value was more pronounced (28.4% control [C] [Fig. 1] to 23.6% sepiapterin-treated [Fig. 3]), indicating that, in agreement with the VC-induced increase in BH4 levels, there was evidence that the vehicle had a small effect on vascular preservation and enhanced vascular coverage. Importantly, in the eNOS-GFP group, BH4 supplementation had a more pronounced impact on the vascular free areas (Fig. 3A, 3B) and reversed the exaggerated response seen in the absence of adequate BH4 levels (34% VC decreased to 23% sepiapterin). VEGF levels were unchanged in all treatments (Fig. 3C).

### Effect of Hyperoxia on BH4 Levels and eNOS Function in RMEC

**Figure 4:**
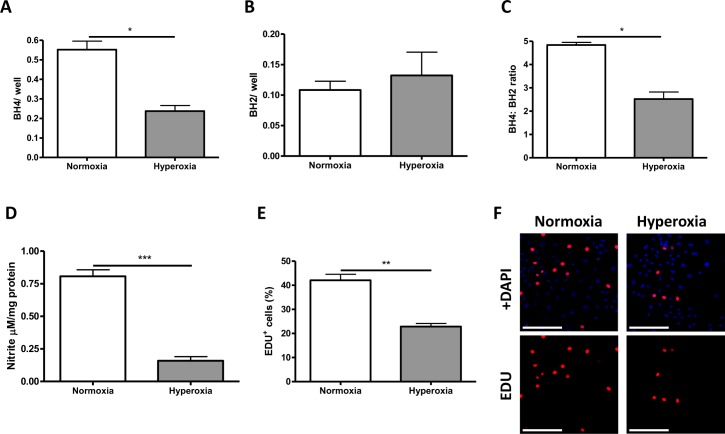
In vitro, hyperoxia depletes BH4 levels, reduces nitrite formation, and impairs the proliferative ability of RMEC. (A) RMECs were cultured under normoxic and hyperoxic conditions for 24 hours and samples were collected and biopterin levels were analyzed by HPLC. BH4 was significantly reduced by hyperoxia exposure, and this was reflected in an increase in (oxidized) BH2. (D) Measurement of nitrite from cells cultured in hyperoxia indicates that there is a significant reduction in nitrite production compared to that in normoxia. Scale bars: 200 μm. (E) Assessment of proliferation by EDU staining indicates that hyperoxia reduces proliferation in RMECs exposed to 75% oxygen. (F) Representative images. *P < 0.05, **P < 0.01, ***P < 0.001.

Next we wanted to investigate the mechanism of the BH4-mediated improvement in vascular preservation in hyperoxia, specifically in RMECs. In cells subject to high oxygen conditions, biopterin levels demonstrated a significant hyperoxia-induced depletion (almost 2-fold) of BH4 (Fig. 4A). BH2 was slightly elevated in hyperoxia compared to that in normoxia (Fig. 4B), resulting in a decrease in the BH4:BH2 ratio (Fig. 4C), suggesting that in hyperoxia, a small percentage of BH4 was oxidized to BH2. These findings correlated with a 3-fold decrease in nitrite levels (Fig. 4D). Hyperoxia had a marginal effect on apoptotic cell death (data not shown); however, the effect was slight compared to the effect on proliferative potential of the cells; EdU positivity was reduced from 42.1% ± 2.5%, expressed as [%EdU-positive/total cell number/well] in normoxia to 22.9% ± 1.3% in hyperoxia (Fig. 4E, 4F).

### Effect of Sepiapterin Supplementation on Hyperoxia-Mediated RMEC Dysfunction

**Figure 5:**
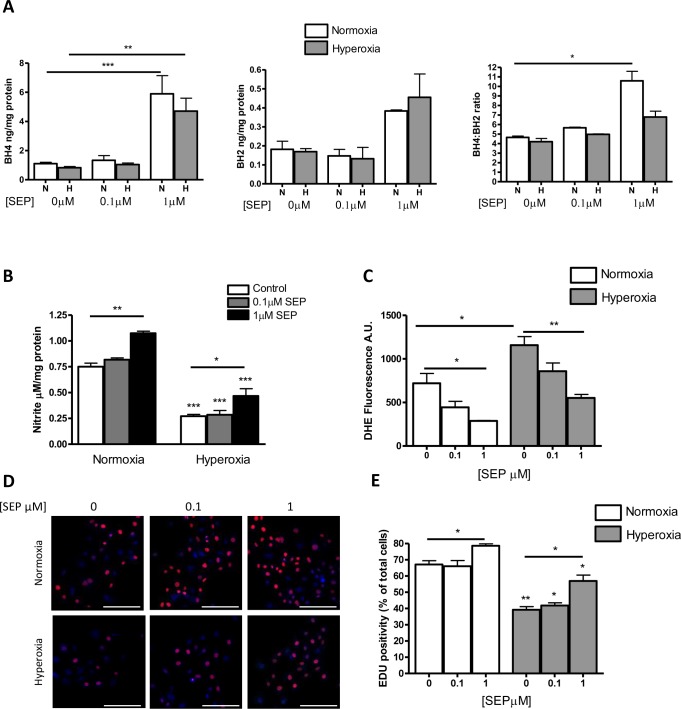
Sepiapterin supplementation improves BH4 levels in hyperoxia, increases production of nitrite, and enhances RMEC proliferation. (A) HPLC analysis of biopterin levels in proliferating RMECs treated with sepiapterin under normoxic and hyperoxic conditions shows that hyperoxia exposure results in reduced BH4, which can be improved by supplementation with sepiapterin. (B) Nitrite measured using the Griess assay following 24 hours hyperoxia exposure. Under hyperoxic conditions, nitrite production in all treatment groups was significantly decreased compared to that under normoxia and increased by sepiapterin. (C) Measurement of superoxide production by DHE indicates that there is a significant increase in superoxide production in hyperoxia, which is reduced by sepiapterin under normoxic and hyperoxic conditions. (D) Immunolocalization of EDU, a marker of proliferation, is shown. Hyperoxia reduces proliferation in RMECs. This can be corrected by addition of sepiapterin to cells (E). Data were derived from n = 3 independent experiments; *P < 0.05, **P < 0.01, ***P < 0.001. Scale bars: 200 μm.

In vitro, using sepiapterin concentrations previously shown to cause a beneficial BH4- dependent increase in NO levels in EC, we showed an elevation of BH4 levels in normoxia (Fig. 5A).^22^ In hyperoxic samples, there was a similar, albeit smaller, increase. The levels of BH2 (Fig. 5B) were increased in a similar manner, indicating some oxidation of BH4 that was consistent in both normoxia and hyperoxia. Calculated relative to each other, there was a reduction in the BH4:BH2 ratio in hyperoxia only at the highest concentration (Fig. 5C). Overall, however, there was a total increase in BH4, demonstrating that the sepiapterin had compensated for the hyperoxia-induced BH4 defect. Notably, compared to cells treated in the absence of vehicle (Fig. 4A), the degree of hyperoxia-induced reduction in BH4 shown in Figure 5A was not as marked, indicting some effect of the vehicle alone.

Next, the consequences of BH4 supplementation on function were determined by nitrite and in situ free radical production and showed that sepiapterin reversed the hyperoxia-induced decrease in nitrite, in line with the improvements in BH4 levels (Fig. 5B, Supplementary Fig. S3). Measurement of superoxide production by DHE fluorescence showed a hyperoxia-induced increase which was markedly inhibited by sepiapterin, especially at the highest concentration (Fig. 5C). DHE fluorescence was inhibited by SOD and L-NAME, indicating NOS was one of the sources of superoxide (Supplementary Fig. S4). In normoxia, the sepiapterin had a small effect on proliferation, an effect likely to be due to the high baseline proliferative rate in these cells (Fig. 5D, 5E). In contrast, sepiapterin had a marked restorative effect on proliferation and reversed the hyperoxia-induced proliferative impairment.

### Effect of BH4 on Retinal EC Proliferation In Vivo

**Figure 6:**
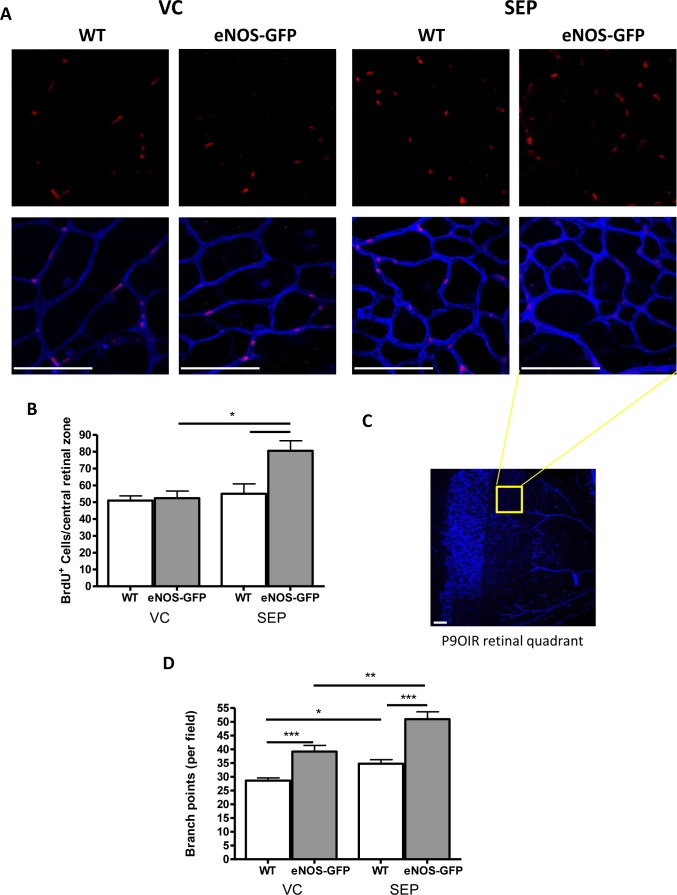
eNOS functional normalization is associated with enhanced EC proliferation during hyperoxia exposure. (A) The proliferation of vascular cells in the central retinal region at the vascular-avascular interface was quantified following hyperoxia by BrdU incorporation. (B) Sepiapterin supplementation caused a significant increase in the number of cells showing BrdU positivity. (C) Low-power image of retinal flat mount indicating area of sampling ([A] yellow). Original magnification is 4×. (D) Retinal vascular branching was also increased following supplementation. Scale bars: 100 μm; n = 3 litters per group. *P < 0.05, **P < 0.01, *** P < 0.001.

The proliferation and branching of vascular cells (Supplementary Fig. S5) at the vascular-avascular interface was quantified following hyperoxia by BrdU incorporation. In line with the improvement in vascular coverage in the supplemented group in the presence of augmented eNOS, there was a marked increase (54%) in eNOS-GFP retinas (Fig. 6). Notably, the largest difference in proliferation in the presence of additional BH4 was observed close to the interface between the vascular and avascular retina. Together, this demonstrated that the BH4-dependent improvement in eNOS activity had a positive effect on proliferation in vivo. Vascular branching was increased in both groups (WT and eNOS-GFP), suggesting some effect of BH4 on the WT group that was not observed in the determination of vascular coverage shown in Figure 3.

## Discussion

In ROP, the stunting of normal inner retinal vascular development has sight-threatening consequences for infants born very prematurely. Current treatments for ROP focus on late disease and are not always effective. An ideal treatment strategy would not only target the initial vasodegenerative stage to preserve EC integrity and prevent capillary regression but also enable continued vascular development during hyperoxia and prevent progression to the sight-threatening neovascular stage of disease. Using loss- and gain-of-function mutants, we and others previously showed that dysfunctional eNOS and eNOS-derived peroxynitrite play an important role in driving vacsular regression in hyperoxia.^7,10^

Our group has also shown that hyperoxia has significant consequences for BH4 bioavailability and eNOS function in the neonatal retina, suggesting limiting BH4 levels as one mechanism responsible for the impaired eNOS function.^10,15^ eNOS has important prorepair functions in normal vascular development and ischemia. Therefore, here our aim was to investigate whether supplementing in vivo with BH4 would reverse the hyperoxia-induced eNOS defect and exacerbated capillary regression and be therapeutically beneficial.

In this study, we report several novel findings and show that the hyperoxia-induced vascular damage observed in the presence of eNOS overexpression is reversed by BH4 supplementation acting, in part, via a reversal in hyperoxia-induced EC proliferative impairment. First, in a transgenic model of endothelium-specific eNOS overexpression, we showed that hyperoxia decreases BH4 levels in retinas of animals. This finding is consistent with our previous study using isolated retinal homogenates where we showed that there was a mismatch between the level of total eNOS expression and enzymatic activity with a concomitant increase in ROS production.^10^ This defect in enzyme function was partly corrected by the addition of BH4 to the homogenate, allowing us to infer that the amount of BH4 was insufficient to supply the demands of the additional eNOS and responsible for the eNOS dysfunction during hyperoxia. Here, in the current study, by specifically measuring BH4 after hyperoxia in the same strain, we showed further evidence for this hypothesis. Moreover, this was further corroborated by the finding that in vivo BH4 supplementation led to a significant improvement in NOS activity, NO bioavailability, and a decrease in hyperoxia-induced oxidative stress and reversed the exaggerated vascular regression observed in this model, consistent with a normalization of eNOS to NO output. Together, this provides evidence that normalizing the cellular redox state has a beneficial outcome in hyperoxia.

With regard to magnitude of effect, there was still some evidence that the redox balance could be improved further. For example, in the hyperoxic eNOS retinas, the BH4:BH2 ratios were slightly lower than those in WT mice following sepiapterin supplementation, suggesting some peroxynitrite-induced oxidation of BH4 to BH2. This was also evident in the NOS activity assay, whereby sepiapterin increased NOS activity to similar levels in both the WT and eNOS-GFP groups. Although it is possible that this might have been due to autoinhibition of eNOS, taken together with the BH4:BH2 ratios, it is also likely to be caused by a higher residual proportion of dysfunctional eNOS in the augmented eNOS group.^28^ The latter would suggest that the amount of bioavailable BH4 is still not optimal or the involvement of an alternative BH4-independent mechanism of eNOS uncoupling, such as oxidative-stress induced glutathionylation of eNOS.^29^ Together, this suggests further avenues for improving vascular outcomes during hyperoxia to maximize the effect of eNOS functional preservation.

Vascular growth in the retina is driven and guided by hypoxia-induced VEGF released from retinal neural cells or astrocytes. In hyperoxia, this relationship is disturbed, and both a decrease in the prosurvival cues of VEGF and a reduction in VEGFR2/Akt signaling are proposed to lead to apoptotic EC death, initiating vascular regression.^2,11^ Significantly, here we show that the effects observed were independent of VEGF levels, which were unaffected by BH4 supplementation, suggesting an EC cell autonomous role for eNOS and BH4 in improved vascular coverage. Thus, second, because ROP primarily affects the vasculature, we showed that hyperoxia specifically depleted BH4 levels in isolated EC with adverse consequences on nitrite production and proliferative potential. Third, we showed that this hyperoxia-induced defect was reversible by increasing BH4 levels, which increased nitrite and importantly also decreased hyperoxia-induced ROS production, indicative of improved NOS function. This positive effect on the cellular redox balance partly reversed the hyperoxia-induced proliferative defect in RMEC, confirming a BH4-dependent mechanism involved in promoting EC growth that is compromised by hyperoxia. Other groups have focused on studying the effect of hyperoxia on apoptosis.^9,11^ Here, we decided to focus on studying the effects of hyperoxia on proliferation, as these effects were much greater than those measured for apoptosis. Thus, fourth, we also showed that, in common with the in vitro results, BH4 supplementation improved the proliferative rate of retinal EC in the eNOS-GFP group in vivo, demonstrating that the vasoprotection afforded by BH4 promoted continued proliferation during hyperoxia.

Taken together, our results here show that a BH4-dependent increase in EC proliferation in eNOS-GFP animals aids in maintaining vascular coverage in hyperoxia. This is in line with previous studies showing a role for eNOS-derived NO in regulating the cell cycle; indeed, the critical role for eNOS in this pathway is evidenced by studies in eNOS^−/−^ mice, which have a significant disruption in the expression of cell cycle genes during collateral vascular development following ischemia.^30^ Diabetic animals also show a NOS-dependent impairment in EC proliferative ability that is reversed by replenishing BH4 levels, restoring their ability to synthesize NO and proliferate more efficiently.^31,32^ Importantly, our studies suggest that this important function of eNOS-derived NO during development is similarly inhibited by hyperoxia and that the enhanced vascular coverage in the BH4-treated eNOS group is due in part to continued proliferation in hyperoxia. Interestingly, the differences in proliferative response and vascular branching were most pronounced close to the interface between the vascular and avascular retina, suggesting that these cells were undergoing continued growth when eNOS function was optimal.

With regard to choice of modulating agent, because BH4 is labile, it would be difficult to administer directly to the retinal circulation. Importantly, sepiapterin, as well as being more stable, had the advantage of being able to cross the blood brain barrier in addition to having good tissue retention properties.^20,23^ Indeed, here we show that sepiapterin is efficiently delivered to the retina and is functionally translated into increased BH4 and a significant improvement in NOS activity. In order to improve the stability of BH4, we used DMSO as vehicle, which interestingly alone showed evidence of having a vasoprotective effect. In this study, there was clear evidence that the vehicle was modulating BH4 levels, suggestive of an antioxidant effect. This was more obvious in the WT group, evident as elevated retinal BH4 levels, reduced NT, and improved vascular area compared to uninjected controls. DMSO has previously been reported to have antioxidant and immunosuppressive effects, to be inhibitory to EC proliferation, and to be antithrombotic.^33–35^ The results of this study suggest that these effects are mediated in part by altering BH4 levels, resulting in a vasoprotective outcome in the retina.

Interestingly, despite evidence of a marked BH4-mediated increase in nitric oxide and NOS activity, BH4 showed only marginal benefit on vascular branching and coverage in WT retinas but had a pronounced positive impact in the eNOS-GFP retinas. Thus, an intriguing aspect of our findings was that, although there was a marked increase in NOS activity following BH4 supplementation, this increase did not yield a better outcome in terms of capillary cover. In contrast, in isolated EC, BH4 had a much more marked effect on hyperoxia-induced EC damage. Together, this suggests a retinal specific or non-EC-derived effect limiting the beneficial outcome of sepiapterin supplementation on vascular coverage. One particular strength of our study was the use of the EC-specific eNOS-GFP animals to manipulate the relative levels of eNOS and BH4 specifically in the endothelium. Taking the in vivo and in vitro results together, our results suggest that a nonvascular/non-EC-derived factor is having an effect on vascular growth. In addition to eNOS, BH4 is also a cofactor for nNOS and the aromatic hydroxylases. Tyrosine hydroxylase is particularly prevalent in early development and is strongly expressed in retinal neural cells; therefore, sepiapterin had the potential to alter the activity of tyrosine hydroxylase.^36^ Indeed, we have previously shown that lower retinal BH4 levels in GTP-cyclohydrolase (GTPCH)-deficient animals correlated with reduced dopamine levels.^15^ Here, sepiapterin did indeed elevate dopamine levels in the neonatal retina to a similar degree in both the WT and eNOS-GFP groups. As a neural tissue, the parenchymal cells of the retina have a significant influence on vascular development: highest during the first week of postnatal week following birth.^37^ During this timeframe, the cross-talk between the tyrosine hydroxylase-positive dopaminergic amacrine cells and the vasculature would be particularly influential. Because dopamine inhibits EC proliferation and is antiangiogenic, BH4-dependent mechanisms would also have a consequence on this pathway.^38^ Indeed, we found previously that GTPCH-deficient hph-1 mice display a decrease in NOS and dopamine levels, which enhanced VEGF levels during development.^15^ With regards to nNOS, we did not observe any evidence of compensatory changes in expression across genotypes as described in eNOS^−/−^ animals.^39^ However it is likely that sepiapterin increased the activity of nNOS similarly in both groups that was evident as an additional increase in total NOS activity above that produced by eNOS. Importantly, there is increasing evidence of neural cell involvement in vascular development; therefore, taking the paradoxical elevation in NOS activity in the WT along with the small effect on vascular coverage, it is possible that neuronal or perivascular nNOS-derived NO is limiting vascular growth, as it does in the brain during a similar developmental window to that investigated here.^39,40^ In the current study, supplemental retinal BH4 levels only had a marginal effect on vascular regression in the WT group. It is therefore possible that the increased dopamine or nNOS activity following sepiapterin supplementation negated the impact of enhanced endogenous eNOS activity in the WT group. The eNOS-GFP group however, allowed us to directly compare the effect of eNOS when BH4 levels were limiting, as was the case during hyperoxia and when supplementary BH4 was sufficient to drive eNOS activity and endothelial proliferation. This is an important mechanism to define, as putative therapies that reduce overall cellular oxidative stress, for example by inhibiting EC-derived eNOS, would also inhibit its prosurvival and reparative functions.

Taken together, our study demonstrates that, in the presence of additional eNOS, the levels of BH4 are insufficient to enable the eNOS-GFP animals to, first, overcome the hyperoxia-induced detriment of BH4 deficiency and, second, to capitalize on the proangiogenic benefits of more functional eNOS activity and that, importantly, supplementation with BH4 can reverse this defect. Previous studies have shown the importance of nitro-oxidative stress as a driver of the VOB phase of OIR and have also shown that peroxynitrite scavengers decrease vaso-obliteration.^7,9^ Here we show, in addition to the decrease in oxidative stress, the benefit of modulating EC-specific BH4 levels to promote eNOS function and facilitate its reparative roles.

With regards to a therapy to prevent ROP, timing of treatment is critical. For example, in line with the differential effects of VEGF in phase 1 (protective) and NV promoting in phase 2, hyperoxia-mediated impairment of VEGFR2 signaling is beneficial in the ischemic phase of OIR, when VEGF activation is maximal.^2,11,41^ Like VEGF in phase 2, prolonged eNOS activation, especially during ischemia, is likely to increase both normal and NV growth; therefore, titrating the benefits of BH4 will be important considerations for future therapies. Importantly, intervening in phase 1 would have the potential to preserve vascular integrity and prevent progression to the sight-threatening NV stage. In this regard, newly approved stabilized BH4, already in clinical use for GTPCH-deficient dystonia, could be used to increase BH4 levels globally.^42^ Our results, however, argue for an endothelial-specific enhancement of BH4. Therefore, treatment strategies would be best used if targeted to the endothelium or in combination with other agents known to protect the retinal circulation in early ROP. This combined approach could, for example, include erythropoietin. Previously shown to be protective against hyperoxia-induced vessel loss in OIR, erythropoietin has also been shown to increase BH4 bioavailability and protect against oxidative stress induced by eNOS uncoupling in the cerebral microvasculature, suggesting a BH4-eNOS–mediated mechanism for its beneficial effects on the retina.^43,44^ Several other agents, notably ascorbic acid and folate, are also known to alter EC-BH4 levels and could therefore be administered safely to premature infants to prevent ROP. In addition to ROP, eNOS function also plays an essential role in diabetes. For example, eNOS knockout animals have a greater range of retinal vascular complications.^45^ Thus, strategies that preserve the prosurvival functions of eNOS would also be beneficial for the prevention of diabetic retinopathy.

In summary, our findings show an important role for BH4 and eNOS in hyperoxia-induced proliferative impairment and vasoretardation in the retina and the positive impact that improving eNOS has on vascular integrity in hyperoxia in the neonate. We also show the validity of using BH4 supplementation to harness the prorepair functions of eNOS in the endothelium and protect against hyperoxic insult. Importantly, this knowledge will aid the development of new strategies to preserve vascular integrity and facilitate normal vascular development in the neonate.

## Supplementary Material

Supplement 1
