# Supplementary material for: BH4-Mediated Enhancement of Endothelial Nitric Oxide Synthase Activity Reduces Hyperoxia-Induced Endothelial Damage and Preserves Vascular Integrity in the Neonate
Source: Invest Ophthalmol Vis Sci. 2017 Jan;58(1):230–41. doi: 10.1167/iovs.16-20523 (PMC6039076; doi:10.1167/iovs.16-20523)
Supplement: Supplement 1 [file iovs-57-15-09_s01.pdf]

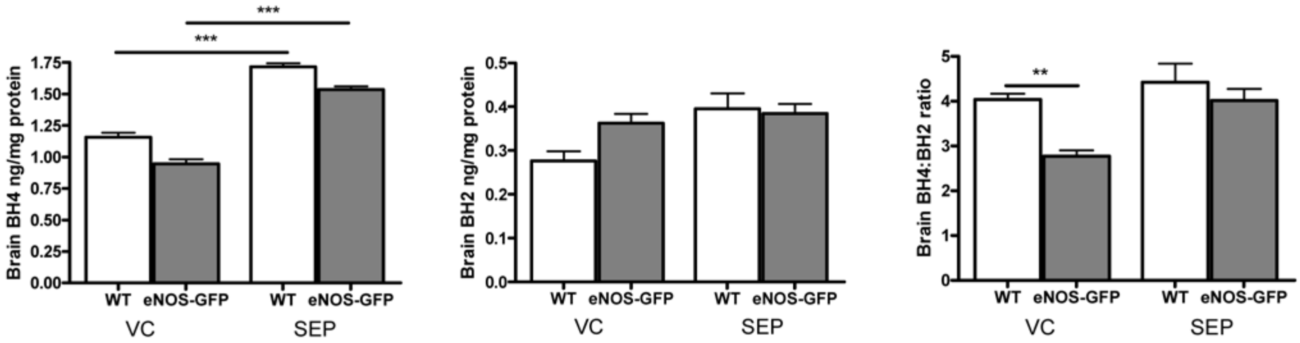

Supplementary Figure S1. eNOS-GFP and WT littermates were injected with vehicle control or 10 mg/kg sepiapterin at P7 before being exposed to hyperoxia for 48 h. Biopterin levels were measured in brain to confirm effect of sepiapterin supplementation on tissue BH4 level. Supplementation with sepiapterin caused in increase in brain BH4 level in both WT and eNOS-GFP mice. There was a significant decrease in BH4:BH2 ratio in the eNOS-GFP mice compared to WT in the untreated group. Histogram shows mean of 3 samples per group. **p<0.01, *** p<0.001.

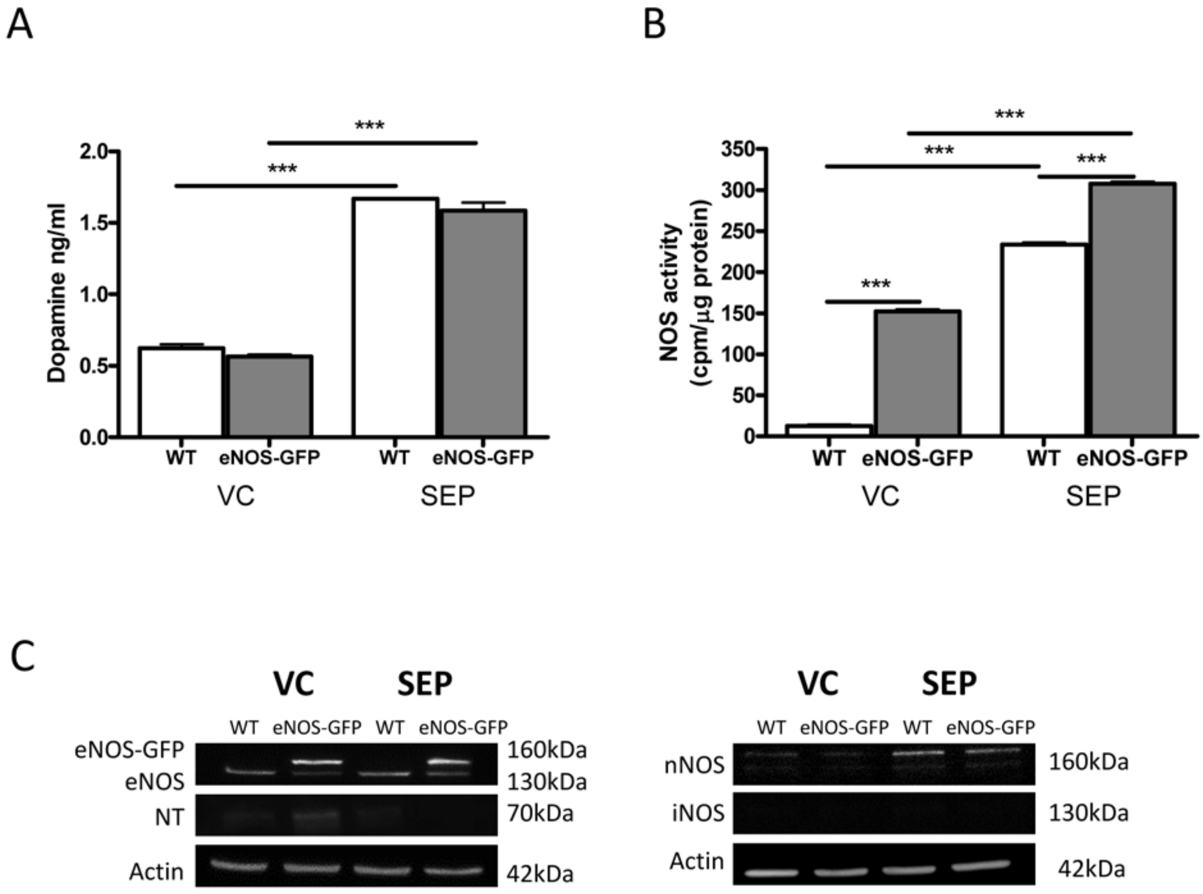

Supplementary Figure S2. A- Retinal dopamine level is not significantly different between eNOS-GFP and WT mice. Following supplementation with sepiapterin from P7 to P9, there is a significant increase in dopamine level in the retinas of both WT and eNOS-GFP mice. B- NOS activity, measured by the conversion of C-14 labelled arginine to citrulline, showed a significantly greater NOS activity level in eNOS-GFP compared to WT retinal lysate. In samples from mice supplemented with sepiapterin, there was a significant increase in NOS activity compared to controls. Part of the pooled retinal samples was analysed by western blot; example blots for eNOS, iNOS, nNOS and nitrotyrosine are shown in C. ***p<0.001.

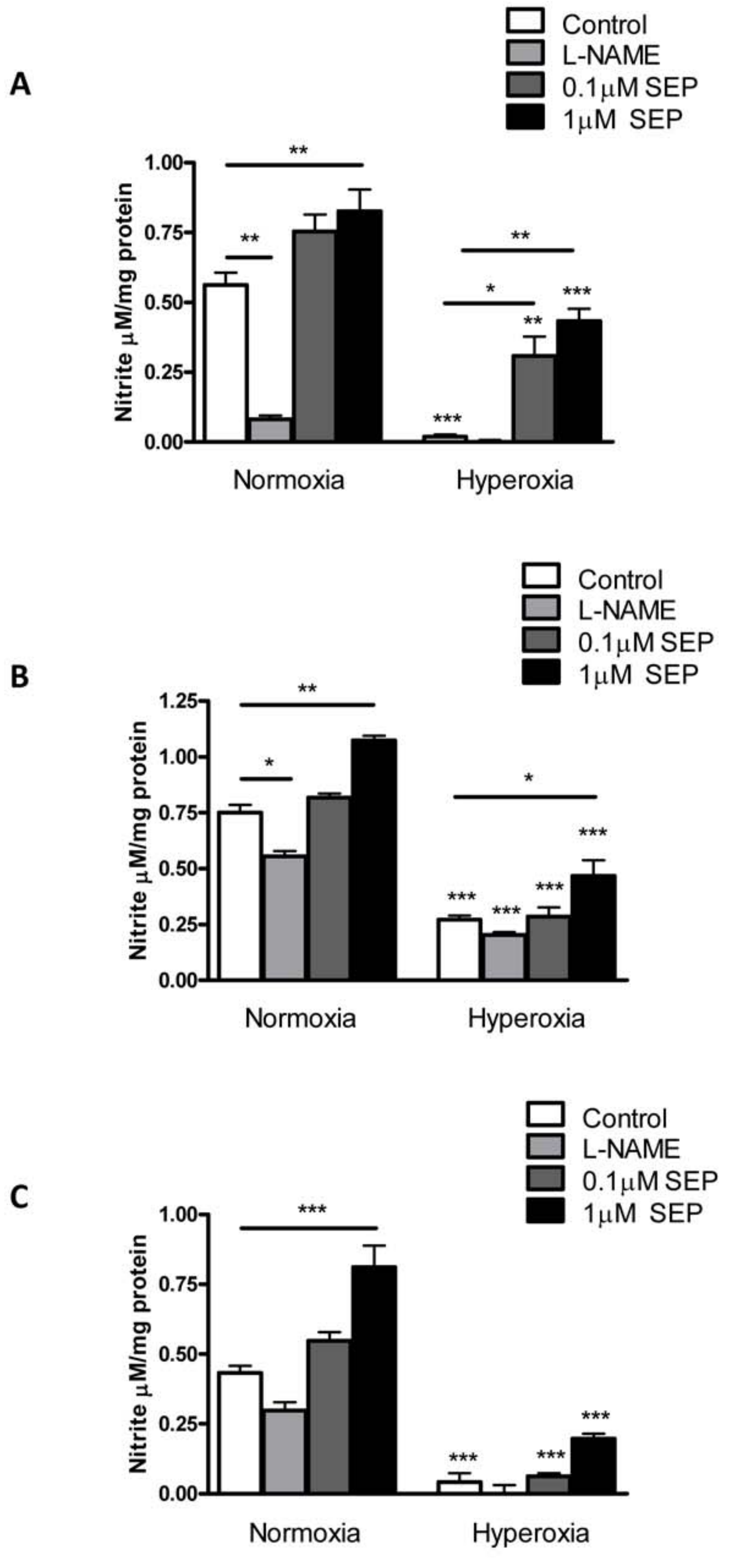

Supplementary Figure S3. Hyperoxia results in a significant decrease in nitrite in RMEC, which is improved by sepiapterin treatment. Nitrite was measured by Griess assay following 24 h hyperoxia exposure. A-C Three independent experiments. *p<0.05, **p<0.01 ***p<0.001.

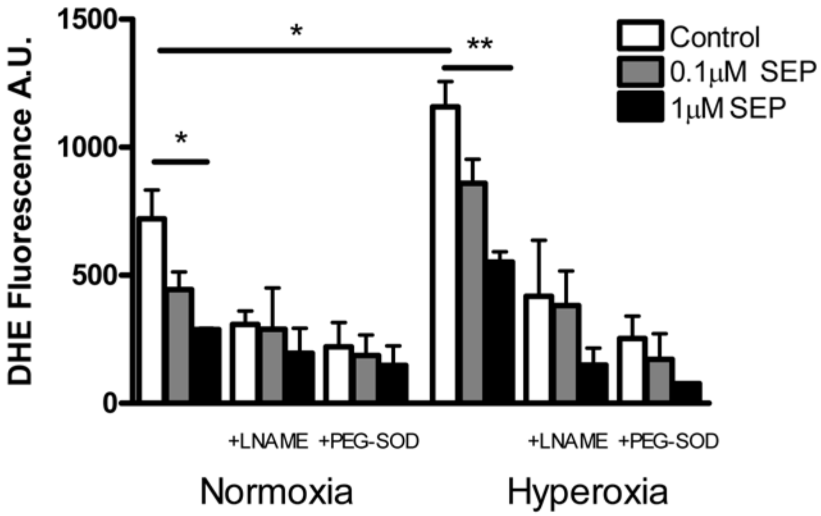

Supplementary Figure S4. Hyperoxia results in a significant increase in DHE fluorescence in RMEC, which can be reduced by treatment with sepiapterin. Incubation of RMEC with L-NAME and PEG-SOD during DHE staining results in a reduction in DHE fluorescence, indicating a role for NOS derived superoxide in contributing to DHE oxidation. *p<0.05, **p<0.01.

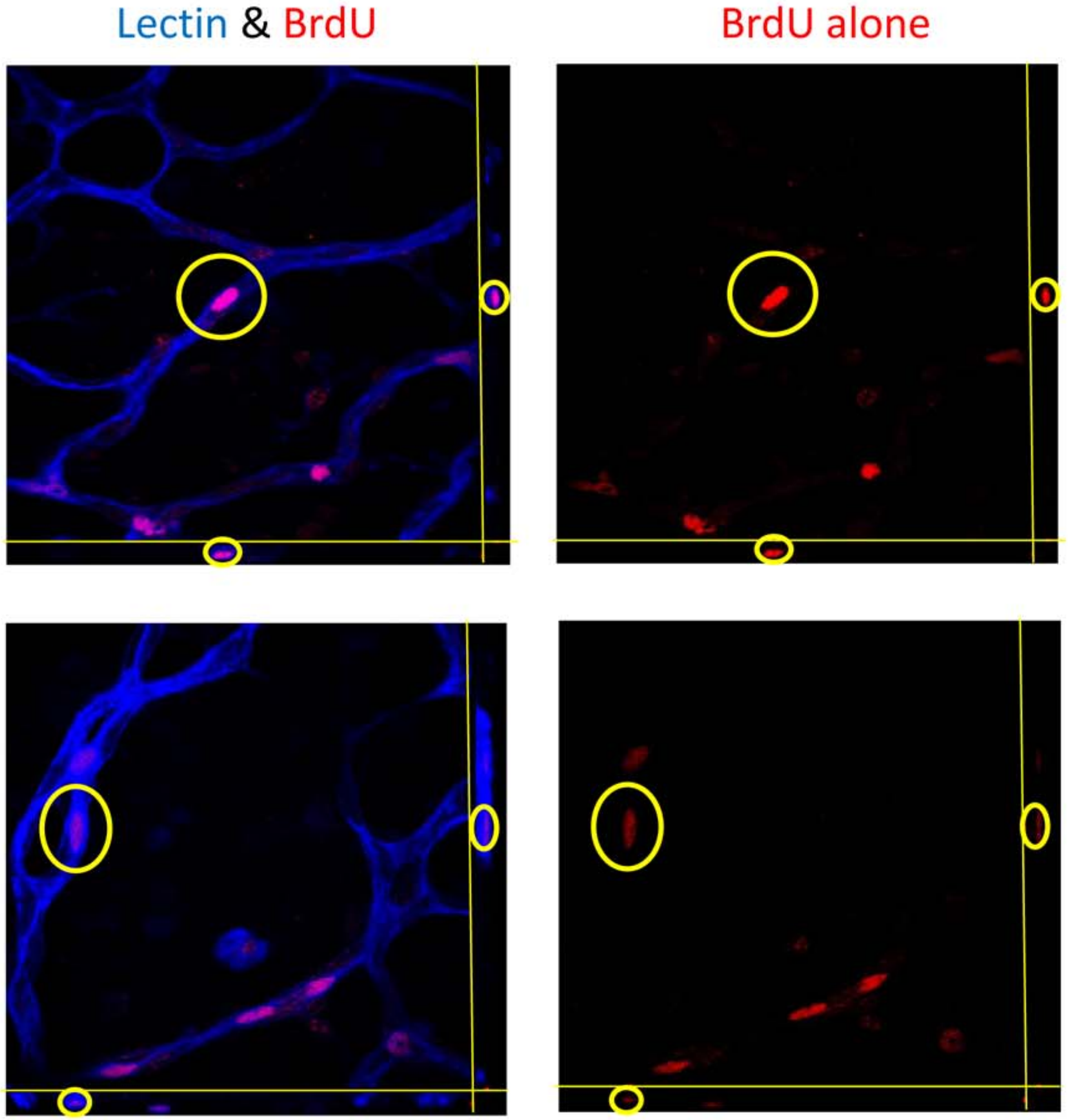

Supplementary Figure S5. Confocal z-series images demonstrating the localization of BrdU positive cells (red staining) in the lectin stained (blue) vascular region. The cell indicated by the yellow circle is also visible in the side panels of the zseries image, in the region to the right and below the yellow lines. These panels indicate that the BrdU positive cell is in the same focal plane as the vasculature.
